# Opinion on the Hurdles and Potential Health Benefits in Value-Added Use of Plant Food Processing By-Products as Sources of Phenolic Compounds

**DOI:** 10.3390/ijms19113498

**Published:** 2018-11-06

**Authors:** Adriano Costa de Camargo, Andrés R. Schwember, Roberto Parada, Sandra Garcia, Mário Roberto Maróstica Júnior, Marcelo Franchin, Marisa Aparecida Bismara Regitano-d’Arce, Fereidoon Shahidi

**Affiliations:** 1 Departamento de Ciencias Vegetales, Facultad de Agronomía e Ingeniería Forestal, Pontificia Universidad Católica de Chile, Casilla 306-22, Santiago, Chile; aschwember@uc.cl (A.R.S.); roberto.paradasalazar@gmail.com (R.P.); 2 Department of Food Science and Technology, Londrina State University, Londrina 86051-990, Parana State, Brazil; sgarcia@uel.br; 3 Department of Agri-Food Industry, Food & Nutrition, “Luiz de Queiroz” College of Agriculture, University of São Paulo, Piracicaba 13418-900, São Paulo State, Brazil; marisadarce@gmail.com; 4 Department of Biochemistry, Memorial University of Newfoundland, St. John’s, NL A1B 3X9, Canada; 5 Department of Food and Nutrition, University of Campinas—UNICAMP, Campinas 13083-862, São Paulo State, Brazil; mmarosti@unicamp.br; 6 Department of Physiological Sciences, Piracicaba Dental School, University of Campinas, Piracicaba 13414-903, São Paulo State, Brazil; marcelo.franchin@yahoo.com.br

**Keywords:** phenolic biosynthesis, microbiological safety, phenolic identification, phenolic bioavailability, phenolic antioxidants, cardiovascular disease, cancer, diabetes, obesity, inflammation

## Abstract

Plant foods, their products and processing by-products are well recognized as important sources of phenolic compounds. Recent studies in this field have demonstrated that food processing by-products are often richer sources of bioactive compounds as compared with their original feedstock. However, their final application as a source of nutraceuticals and bioactives requires addressing certain hurdles and challenges. This review discusses recent knowledge advances in the use of plant food processing by-products as sources of phenolic compounds with special attention to the role of genetics on the distribution and biosynthesis of plant phenolics, as well as their profiling and screening, potential health benefits, and safety issues. The potentialities in health improvement from food phenolics in animal models and in humans is well substantiated, however, considering the emerging market of plant food by-products as potential sources of phenolic bioactives, more research in humans is deemed necessary.

## 1. Introduction

Phenolic compounds exist in their monomeric, oligomeric and polymeric forms. Gallic and ellagic acids are found in plant food and their processing by-products as simple phenolics as well as monomeric units of ellagitannins, also known as hydrolysable tannins. Likewise, catechin and epicatechin exist as simple phenolics but are also constituents of proanthocyanidins (condensed tannins). Oligomeric phenolics possess a degree of polymerization (DP) ranging from 2 to 10 while polymers show DP > 10. Conjugated phenolics and their corresponding aglycones are well known and the latter shows higher hydroxylation degree than that of the conjugated form. Furthermore, monomeric phenolics possess one or more aromatic rings bearing one or more hydroxyl groups while oligomeric and polymeric phenolics have more than one hydroxylated aromatic ring.

Several classes of phenolic and polyphenolic derivatives have been described in the literature [1]. The terms “phenolics” and “polyphenolics” have sometimes been used interchangeably [2]. Phenolic compounds are commonly divided into non-flavonoids and flavonoids, the latter class is most often encountered in the food sources [3]. These chemical bioactives are plant food secondary metabolites and are primarily related to the plant defense against biotic and abiotic stress, pests and pathogens [2,4]. However, studies in vitro, as well as in animal models and humans, also provide support for their potential health benefits by lowering the risk and/or preventing the onset of chronic ailments.

The existing literature shows a myriad of chemical and/or biochemical mechanisms by which polyphenols may be render their effects [5,6,7,8]. Their roles as antioxidants, scavengers of reactive oxygen species (ROS), reducers or chelators of metals ions and in restoring antioxidant enzymes has been well established. Furthermore, inhibitory effects of phenolics towards enzymes related to metabolic disorders such as type 2 diabetes and obesity (e.g., α-amylase, α-glucosidase, and lipase) have also been highlighted. In addition, polyphenols may render anti-inflammatory and antimicrobial effects [9,10,11,12]. Therefore, some authors have suggested that phenolic compounds are perhaps the most important non-nutrient bioactive compounds in the human diet [2].

The potential of plant food by-products as a source of phenolic compounds has been widely recognized. In particular, by-products from cereals, nuts, oilseeds, fresh and dried fruits, vegetables, spices, coffee, and tea, among others [1,13,14], may be richer in different bioactive phenolics than those of their original sources. Additionally, a recent study has demonstrated that, due to their higher phenolic contents, winemaking by-products are able to better decrease very low-density lipoprotein (VLDL) cholesterol and triacylglycerol levels than those of red wine in vivo [15].

**Figure 1:**
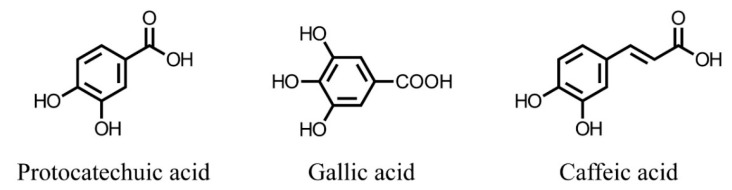
Chemical structures of major phenolic acids identified in peanut skin [12,17] and grape by-products [16,30].

Peanut skin and grape by-products, for example, are rich in proanthocyanidins A and B, also known as condensed tannins [8,16,17,18], whereas pomegranate peels and seeds are rich in hydrolysable tannins (ellagitannins) [19,20,21]. Citrus by-products have a high concentration of low molecular weight flavonoids [22,23], and by-products from blueberry and other emerging berries such as jaboticaba (*Myrciaria jaboticaba* (Vell.) Berg) and juçara (*Euterpe edulis* Mart.) are abundant in anthocyanins [24,25,26,27]. Meanwhile, phenolic acids are prominent in wheat and other cereal by-products [28,29]. The basic structures of common phenolic acids are shown in Figure 1.

Based on the existing knowledge, it is evident that the consumption of different sources of phenolic compounds is of much importance for a better quality of life. This is especially true when it comes to the consumption of edible plant foods and their processing by-products. Furthermore, as an inexpensive alternative source of important biomolecules, plant food by-products may find better uses in the field of functional ingredients and/or nutraceuticals. The distribution of food phenolics among different tissues is influenced by genetic pathways, being specific to each crop and/or variety thus influencing their response to biotic and abiotic stresses in the field, including the production of secondary metabolites. Safety issues of plant food by-products, which must be addressed before application as functional ingredients or in producing nutraceuticals, may differ among feedstocks. Furthermore, storage and food processing may influence their safety parameters. The profiling and screening of phenolics are crucial to anticipate their potential health benefits. Finally, non-communicable diseases (NCDs) such as cardiovascular ailments, cancer, diabetes and obesity, as well as oxidative stress and inflammation are common to all of these chronic effects. In this sense and, considering the importance of plant food by-products as emerging sources of phenolic compounds, the present review summarizes the hurdles and the most promising applications with a special emphasis to their potential health benefits.

## 2. Role of Genetics on the Distribution and Biosynthesis of Plant Phenolics

As already mentioned, plant food by-products are often more abundant sources of phenolics than their corresponding starting materials and/or food products [5,15,31,32]. Thus, it is frequently recommended to consume whole foods and eat certain fruits with their peels. These peels and other processing by-products are not only rich sources of dietary fibre and minerals [33,34,35,36], but are also important sources of phenolic compounds [25,37,38]. Various phenolic compounds, such as phenolic acids and flavonoids, are present in many seeds, particularly in their hulls or seed coats [39]. The role of phenolic compounds in plants is summarized in Table 1. The higher concentration of phenolic compounds in the outer layers of grains and seeds [5,40,41,42,43,44,45] is in part explained by the plant defense mechanisms against pests and pathogens [4]. As such, these phytochemicals are also known as phytoalexins. The greater concentration of phytoalexins in the peels and skins of plant foods is related to their environmental adaptation; as these parts are more exposed to pests and microorganisms than the inner part.

**Figure 2:**
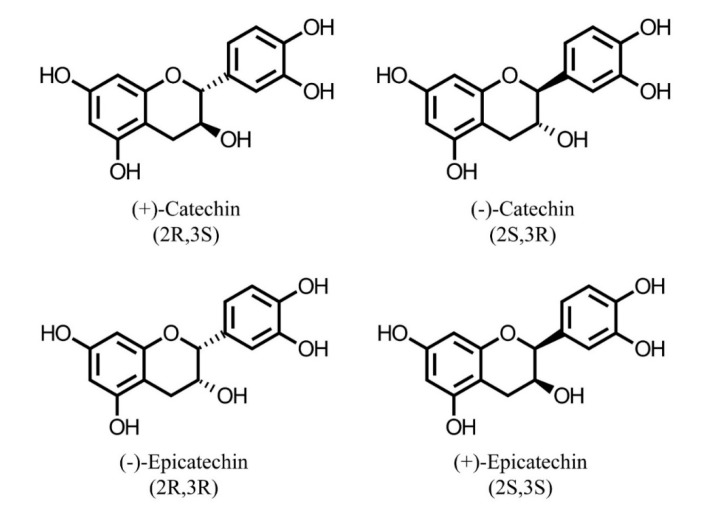
Chemical structures of isomers of monomeric units of procyanidins.

A wealth of data from the literature demonstrates that different parts of the plant contain specific phenolics [12,19,40,80,81], thus suggesting that their accumulation is mediated by particular transcription factors during their biosynthetic pathway. The skins of grape are abundant in flavonoids and phenolic acids while flavonoids are mainly concentrated in the seeds [82]. Other studies have also demonstrated that phenolic acids are constituents of peanut skin and meal from dry-blanched peanuts, whereas proanthocyanidins and monomeric flavonoids are found only in peanut skin [12,31]. The chemical structures of isomers of monomeric units of procyanidins are shown in Figure 2. Furthermore, only four anthocyanins were found in the seeds of pomegranates compared to the 12 identified in the edible part. Meanwhile, proanthocyanidins, which were not detected in the mesocarp and divider membrane, were present in the outer skin of pomegranates [19]. Thus, it is evident that most plant species possess complex mixtures of phenolic compounds, and the concentration and identity of these molecules can vary from organ to organ, and in the different developmental stages of the organism [83]. However, different responses to environmental conditions and stresses also play an important role in the plant composition.

**Figure 3:**
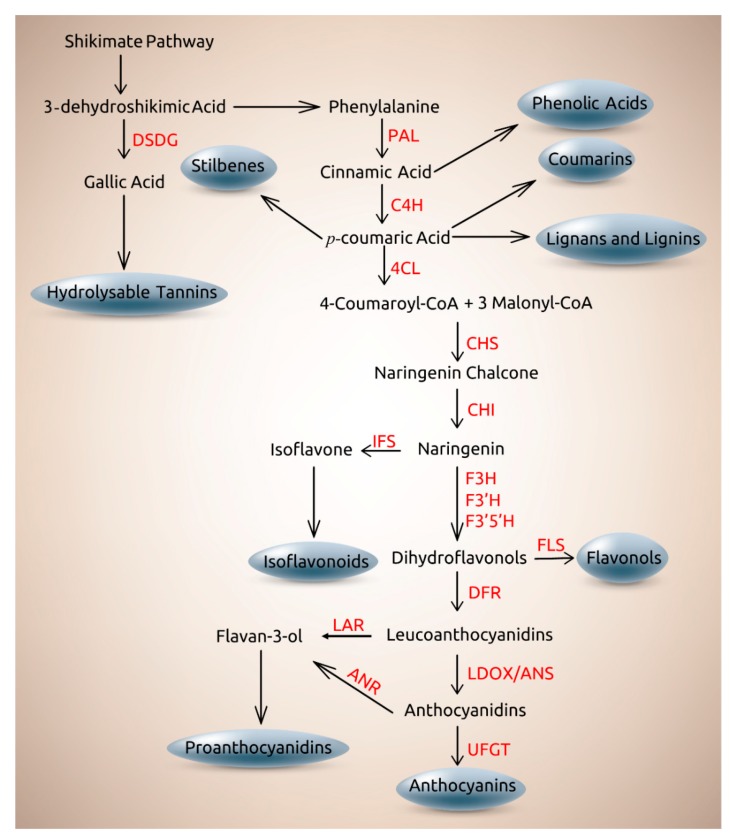
Adapted from the literature [56,89,90,92,93,94,95]. DSDG, dehydroshikimate dehydrogenase; PAL, phenylalanine ammonia-lyase; C4H, cinnamate 4-hydroxylase; 4CL, 4-coumarate:CoA ligase; CHS, chalcones synthase; CHI, chalcone isomerase; F3H, flavanone 3-hydroxylase; F3′H, flavonoid 3′-hydroxylase; F3′5′H, flavonoid3′,5′-hydroxylase; IFS, isoflavone synthase; FLS, flavonol synthase; DFR, dihydroflavonol 4-reductase; LAR, leucoanthocyanidin reductase; LDOX/ANS, leucoanthocyanidin dioxygenase/anthocyanidin synthase; ANR, anthocyanidin reductase; UFGT, UDP-glucose-flavonoid 3-O-glucosyl-transferase.

Plant phenolics are synthesized by two different metabolic routes, the malonic acid and the shikimic acid pathways, converging into the phenylpropanopid pathway [46,84]. The shikimic acid pathway produces aromatic compounds in plants, such as the amino acids tyrosine, tryptophan and phenylalanine [85], but also produces gallic acid as an intermediate compound, which is the precursor of hydrolysable tannins, gallotannins and ellagitannins [86,87,88]. This differs from condensed tannins (proanthocyanidins), synthesized in the flavonoid pathway, derived from the phenylpropanoid pathway [89]. The first reaction in the phenylpropanoid metabolic route is the deamination of phenylalanine by the action of phenylalanine ammonia-lyase (PAL) yielding cinnamic acid and ammonia. Subsequently, cinnamate 4-hydroxylase (C4H) catalyzes the cinnamate hydroxylation into 4-coumaric acid (*p*-coumaric acid). The enzyme 4-coumarate:CoA ligase (4CL) catalyzes synthesis of the CoA thioester 4-coumaroyl CoA (*p*-coumaroyl CoA), which is ATP-dependent [84]. From this point onwards, the different types of plant phenolics are synthetized (Figure 3) [90].

The biosynthesis of flavonoids in plants is well established, with six main types of flavonoids (chalcones, flavones, flavonols, flavandiols, proanthocyanidins and anthocyanins). These compounds are found in most higher plants, and isoflavonoids are mainly present in legumes [89]. The first reaction in the flavonoid branch is catalyzed through the action of chalcones synthase (CHS), where 4-coumaroyl-CoA is combined with 3 malonyl-CoA molecules, obtaining naringenin chalcone, which is converted to naringenin by the action of chalcone isomerase (CHI), being this compound the principal point from which all classes of flavonoids branch out [91]. Further steps including the activity of flavonoid 3-*O*-glucosyltransferase, dihydroflavonol 4-reductase, flavonoid 3′-hydroxylase, and flavanone 3-hydroxylase, among others, generate the rest of the flavonoid groups, as summarized in Figure 3 [56,89,92,93,94,95].

According to Holton and Cornish [92], the study of the genetics of anthocyanin synthesis began last century with Mendel’s work on the pea flower colors. From this point onwards, an extensive amount of experimentation has been conducted to decipher the genetic basis of pigment synthesis using multiple plant species, standing out the attraction of pollinators and seed dispersal as a consequence of anthocyanins synthesis in petals.

The isolation and characterization of mutants involved in the pigmentation of the aleurone layer and the seed coat have strengthened the genetics and the molecular understanding of this trait in several plant species [96,97]. Specific proteins regulating anthocyanin accumulation have also been characterized and studied in detail in several plant species [98,99]. These proteins are included in the two biggest families of plant regulatory proteins, the bHLH and the MYB families [100,101,102,103]. Other plant proteins carrying the “WD40” repeats (WDR or beta-transducin repeat) are also implicated in the expression of pigmentation [104,105,106].

The MYB component of the MBW (MYB-bHLH-WDR) complex activates this pathway through the transcription of its bHLH partner, and the MBW complex is considered a “master regulator” that can stimulate this pathway by itself [107,108,109]. More than 600 types of anthocyanins have been reported to date [110], and after synthesis, they are transported to the vacuolar lumen where they are stored [111,112]. There is agreement that the MBW complex controls a series of regulative proteins distinctly from a highly organized transcription process depending on specific environmental conditions to the beginning of the flavonoid biosynthetic route through a positive regulatory feedback [113]. These types of environmental and developmental controls mainly depend upon the well-orchestrated expression of early biosynthetic genes (EBGs) and late biosynthetic genes (LBGs). At least 17 genes in *Arabidopsis* that control the flavonoid (flavonols and proanthocyanidins) metabolism during seed development have been reported [114]. Recently, Xu et al. [115] demonstrated that the transcription factors TRASPARENT TESTA 16 (TT16) and 15 (TT15) work upstream the proanthocyanidin biosynthetic pathway, although through two different genetic pathways that control proanthocyanidins accumulation in *Arabidopsis* seed coats. In this context, six of the *tt* genes have been reported to encode transcriptional regulators, which are, TTG1 (WDR family), TT1 (WIP1/Zn finger), TT16 (ABS/AGL32, MADS box), TT8 (bHLH042), TT2 (DSL1/WRKY44) and TT2 (MYB123), standing out the relevance of transcriptional controls in the regulation of flavonoid biosynthesis [114]. TTG2, TT1, and TT16 are also implicated in epidermal cell fate, which can be an indirect way to regulate proanthocyanidin accumulation [113]. The control of flavonol biosynthesis encompasses distinct R2R3-MYB transcription factors aiming EBGs and flavonol synthase (*FLS*), specifically MYB11, MYB12, and MYB111 [116].

Anthocyanin biosynthesis in *Solanaceous* plant species such as pepper, tomato, eggplant, potato and petunia, is controlled by MBW complexes involving different MYBs, although with the same bHLH and WD40 transcription factors. Diminished biosynthesis is regulated through the upregulation of MYB repressors and the downregulation of MYB activators [56]. In radish, total anthocyanin associated with the transcription levels of anthocyanin biosynthesis genes namely, *RsCHS3*, *RsUFGT*, *RsANS*, *RsF3′H1* and *RsF3H*, playing these genes key functions in spatial-temporal and phenotypic anthocyanin accumulation by a coordinated control, and the principal regulatory element in anthocyanin biosynthesis is *RsUFGT* [117]. According to another study, 13 structural genes are likely involved in the anthocyanin biosynthesis in the taproots of purple carrot genotypes, *LDOX1/LDOX2*, *DFR1*, *F3H1*, *F3′H1*, *CHS1*, and *CHI1* genes that can be responsible for the loss of light-independent anthocyanin synthesis of non-purple carrots [94]. In addition, the expression of *LDOX2*, *DFR1*, *PAL1*, *PAL3*, and *F3H1*, which are anthocyanin biosynthetic genes, augmented as a result of an ethephon application in black carrot roots, as well as the expression of the *MYB1* transcription factor, which under stressful conditions was related to the stimulation of the phenylpropanoid pathway [118]. Interestingly, the transient and stable transformation results showed that *IbMYB1* by itself was sufficient to activate all the structural anthocyanin genes and the accumulation of anthocyanins in the flesh of sweet potato roots [119]. In this species, a MADS-box gene (*IbMADS10*) is implicated in pigmentation, resembling that of the *Arabidopsis* transparent testa (*tt*) genes. In another study, contrary to the complex nature of pigmentation (i.e., polygenic inheritance and strong effect of the environment) reported previously, the “white” phenotype of pomegranate was caused by a recessive single-gene trait, due to the insertion in the *Leucoanthocyanidin dioxygenase* (*PgLDOX*) gene, determining the white anthocyanin-less visual aspect [120]. Liu et al. [56] proposed that environmental stimuli involving high light intensity [121], blue/UV light [122] and low temperature [123] are useful during cultivation to stimulate anthocyanin production as a short-term enhancement. For a long-term improvement, modern breeding tools (genetic engineering) can be utilized to not only augment yields, but also to optimize anthocyanins content by the stabilization of their structures and the reduction of their degradation.

Once oxidized, proanthocyanidins generate mature seeds that are brown-colored [124], and they have a pivotal role in the seed embryo protection against abiotic and biotic stressful conditions [97], and in the articulation of seed dormancy, dispersion and longevity [125,126,127]. Apart from their role in seeds [128], proanthocyanidins in leaves provide protection against biotic and abiotic stress, they confer astringency and flavor to wines and other drinks, they provide positive effects for human health, and they are a major quality factor for forage crops [115,129]. In this context, an *Arabidopsis* TT2-like gene *MYB115* was identified in *Populus tormentosa* (poplar) and studied and characterized using several genetic and molecular methods (including CRISPR/Cas9 system), providing knowledge of the regulatory systems controlling proanthocyanidins synthesis through the activity of MYB115 in poplar, improving resistance to fungal pathogens [103]. In parallel, a quantitative trait loci (QTL) mapping and an association analysis were conducted on grape berry proanthocyanidins composition revealing an intricate genetic regulation for proanthocyanidin traits and distinct genetic architectures between skin and seeds, although this study unraveled novel genomic regions (four candidate genes *VvMYBPA1*, *VvCHI1*, *VvMYBPA2*, and *VvLAR1*) that are valuable for future research of the genetic regulation of proanthocyanidins content [130]. Regarding forage crops, Paolocci et al. [131] reported that *FaMYB1* expressed in *Lotus corniculatus* leaves, which is a flavonoid *R2R3MYB* repressor from strawberry, can compensate the activity of the endogenous transcriptional MYB-bHLH-WD40 (MBW) complex stimulating proanthocyanidin synthesis [113,114,132], and *FaMYB1* did not alter the expression of a resident *R2R3MYB* promoter of proanthocyanidins. This study concluded that there is a commitment in leaf cells to produce proanthocyanidins that depends on the balance between the activity of promoter and repressor MYBs working within the MBW complex of forage legumes. In addition, Escaray et al. [133] produced a *Lotus corniculatus* × *L. tenuis* interspecific hybrid that displayed high biomass yield, rhizome production, and elevated proanthocyanidin content in edible tissues adequate to avoid ruminal bloating. This study showed that proanthocyanidin levels correlated with the expression response of the *R2R3MYB* transcription factor TT2, and with those of the essential structural genes of the catechin and epicatechin biosynthetic routes resulting in proanthocyanidin biosynthesis.

In barley grains, the flavonoid biosynthetic pathway has been investigated in detail [134], and the yellow color is caused by proanthocyanidin produced in the seed coat [135]; red and purple pigments are anthocyanins produced in glumes and pericarp; and blue colors are due to anthocyanins produced in the grain aleurone layer [136]. More recently, 11 structural and regulatory genes controlling spatial and temporal responses have been reported, in which the *Ant2* gene plays a crucial role in barley grain pericarp pigmentation, and considering the flavonoid biosynthesis pathway genes, there was a lack of specific transcriptional regulation in black-grained genotypes [93]. One recent genome-wide association study of barley under varying plant water regimes showed that drought had a slight negative effect on the concentration of total phenolics (TP), and five specific TP-related QTLs were identified, which can have great potential for the molecular breeding of barley varieties with improved straw quality for bio-energy applications [137].

Sensory characteristics (e.g., color, texture, and flavor) of plant foods are perhaps the most important factors dictating a consumer’s decision. Furthermore, functional claims may also affect their final price. The content of phenolic compounds as well as their identities play a major role in both sensory and potential bioactivities of plant foods and, as a consequence, their processing by-products. Therefore, current breeding investigations should consider phenotypic responses in terms of (poly)phenol contents and their identities [138]. For example, the choice of a proper genotype is essential for obtaining onions with high-flavonoid content (i.e., red over yellow and white cultivars). In addition, the inedible dry skin has higher total flavonoids relative to that of the edible flesh [139]. Ciancolini et al. [140] selected two genotypes, out of 17 Italian globe artichokes, as the most appropriate source materials to recover bioactive phenolics (e.g., chlorogenic acid and dicaffeoylquinic acid) [140]. However, comparing to other areas such as food processing and their outcome in terms of phenolic changes, it is still necessary to gain a better understanding about the new genetics and plant breeding approaches with respect to the expression and over-expression of genes associated with the biosynthesis of phenolic compounds that are beneficial to human health and possess antiaging activity [141,142,143].

In summary, the genetic material of the plant does not differ within plant tissues. In contrast, the distribution of phenolic compounds among plant tissues is often distinct. Due to the crucial role of several enzymes during the phenolic biosynthesis, it is possible to state that these enzymes may be tissue specific. Therefore, plant breeders could pay special attention to the expression of genes related to production of specific enzymes in order to obtain plant materials that may render by-products enriched in bioactive phenolics. Likewise, in depth understanding of the role of genetics in the distribution of phenolic compounds in distinct forms (e.g., soluble (free, esterified, etherified) versus insoluble-bound) as well as in their monomeric, oligomeric, polymeric, aglycone and conjugated state appears to be a promising field of investigation which will be helpful for better understanding of the potential uses of plant food by-products as sources of these natural compounds.

## 3. Microbiological Safety and Decontamination

Peanuts or groundnuts have their skin removed, if subjected to the blanching process. Several reports have substantiated the role of the skin as a major source of phenolic compounds [12,17,18,31,144,145]. Due to constant contact with the soil and post-harvest conditions, peanuts and their skins may not fit microbiological standards for use in producing nutraceuticals or as a functional ingredient [17,146]. Although peanut skin is used as an example here, the same concept may be extended to different plant food by-products, especially those generated from processing of certain fruits, nuts, grains, seeds, and other non-perishable food, for which storage conditions may not be adequately considered by the producers and the industry. According to Toledo et al. [36], the addition of passion fruit peel and seed flour increased the growth of yeast and mold in a food model system. Brazil nut skin and hard shell, by-products of the cracking and shelling process, have also been regarded as potential sources of bioactive compounds [80], but their safety requires attention. Shelled Brazil nuts from two different locations showed higher aflatoxins than that of in-shell samples [147], lending support to the probable higher concentration of mycotoxins in the shell. Furthermore, bran and shorts showed higher deoxynivalenol concentration compared to the flours [148], also indicating that the outer layers of wheat are more susceptible to mycotoxin contamination. In addition, apples and their products, especially from organic growing, may be contaminated with patulin [149,150], thus, apple peel, a flavonoid-rich by-product [151] may also be contaminated. Therefore, microbiological and/or toxicological status of plant food by-products should be checked and strategies to prevent contamination and to manage their quality standards be contemplated.

Ionizing radiation and ultraviolet radiation have long been used to inhibit or eliminate microorganisms (bacteria and fungi) in food products [146,152,153,154]. However, due to induced free radical generation, detrimental effects towards vitamin C and liposoluble compounds, such as tocopherols and carotenoids, have brought about a concern regarding their effects towards other phenolic compounds [155,156]. The literature, however, has demonstrated that induced changes are dependent on the nature of the compounds involved. Anthocyanins have been found to decrease upon gamma-irradiation [157], but proanthocyanidins, monomeric flavonoids, and phenolic acids increased in the fraction containing free and insoluble-bound phenolics [17]. Although gamma-irradiation may induce negative effects on anthocyanins, the same changes have also been observed upon pasteurization [158]. These methods have been used to decrease the microbial load in the food, cosmetic and pharmaceutical industries, but gamma-irradiation has been found effective not only towards bacteria but also against their toxins [153], which is not the case for heat treatment, in which enterotoxin A has been found to be resistant [159]. In addition, a recent study demonstrated that ozone treatment reduced deoxynivalenol and zearalenone contamination in wheat bran [160]. Several other non-thermal technologies such as pulsed light, high-power ultrasound, cold plasma, high hydrostatic pressure, and dense phase carbon dioxide have been tested to improve the safety of edible products [161], but experience has demonstrated that most of them may induce changes in the identity or in the quantities of phenolic compounds.

## 4. Characterization of Phenolic Compounds

### 4.1. Sample Preparation and Phenolic Extraction

Sample preparation is a key step for qualitative characterization and quantitative analysis of plant food phenolics. Furthermore, there are several types of plant food by-products (e.g., skins, seeds, leaves, bran, etc.), all of which may have different structural moisture contents. Therefore, to facilitate comparison, the final results should be reported on a dry weight basis. Three dehydration techniques were evaluated by Barcia et al. [162], namely oven-drying at 50 °C; spray-drying; and freeze-drying. Regardless of the sample (skins or lees), an examination of the phenolic composition of winemaking by-products (BRS Violeta cultivar) demonstrated that oven-drying negatively affected their concentration. The content of anthocyanins plus pyranoanthocyanins was 18 times lower in oven-dried samples compared to that of freeze-dried samples. The same trend was observed for flavonols, hydroxycinnamic acid derivatives, and condensed tannins, although to a lesser extent. Likewise, the same study showed that stilbenes of winemaking by-products from BRS Lorena cultivar were also negatively affected. Therefore, especially when it comes to anthocyanin preservation, oven-drying should be especially avoided.

Several plant food by-products may also contain significant amounts of lipid in their composition. Fibre is a major constituent of peanut skin [145], however, another study [163] demonstrated that peanut skin still has a significant lipid content (11%). In addition, fruit seeds, well known and investigated processing by-products, are also rich sources of specialty oils [164] containing up to 80% polyunsaturated fatty acids [165]. The content of their unsaturated fatty acids was correlated with their concentration of liposoluble antioxidants [164], such as tocols (tocopherols and tocotrienols), and carotenoids [165]. It is therefore, evident that tocopherols and tocotrienols as well as carotenoids are present in the lipid fraction. Thus, due to their reducing and/or free radical scavenging properties [165], these bioactive compounds may also interfere in different assays. In fact, Arranz et al. [166] reported that DPPH (2,2-diphenyl-1-(2,4,6-trinitrophenyl)hydrazyl) radical scavenging activity was significantly and positively correlated with the antioxidant stability of several nut oils as evaluated by the Rancimat method, which was attributed to their tocopherol contents. The same study also demonstrated that phospholipids interfered in the determination of total phenolic contents by Folin and ortho-diphenols assays. Several solvents have been used to extract polyphenols (e.g., methanol, ethanol, acetone), and these are also able to extract the lipid fraction. Peanut skin extract obtained upon hexane extraction [167] was not able to delay soybean oil oxidation in the Rancimat test in various concentrations (100–800 ppm). In contrast, extracts (100 ppm) obtained with ethanol decreased the induction period of refined-bleached-deodorized soybean oil and showed to be as effective as butylated hydroxytoluene (BHT), thus indicating that ethanol, but not hexane, was able to extract phenolic antioxidants from peanut skin. Therefore, during defatting, which is a mandatory step, one must consider these differences and hexane appears to be the best option thus far.

More than three decades ago, Krygier, Sosulski, and Hogge [168] and subsequently Naczk and Shahidi [169] suggested a successful alkaline extraction method for quantitative extraction of insoluble-bound phenolic acids. In addition, some recent reports have also supported the advantages of this method in recovering monomeric flavonoids, proanthocyanidins, and hydrolysable tannins [16,17,21,81,170]. However, even to date, most studies on phenolic compounds only consider the fraction containing soluble phenolics, ignoring the insoluble-bound fraction, which is linked to the cell wall of the plant material. Furthermore, the fraction containing soluble phenolics, also known as crude phenolic extract [171], may also be fractionated into free and soluble-conjugated molecules, namely esterified and etherified phenolics [31]. Fractionation techniques have been shown to be helpful for the identification of new phenolic compounds, which allows deeper evaluation and may help to enrich the phenolics database with respect to the evaluation of a crude extract [31]. In fact, 79 phenolic compounds in different parts of pomegranate by-products have been reported, from which 30 compounds were identified for the very first time [21]. Furthermore, proanthocyanidins, reported in pomegranate by-products for the first time, were present mainly as soluble conjugates in the fraction containing phenolics released from their esterified form. This indicates that proanthocyanidins were esterified with other molecules and their identification could be very difficult without prior hydrolysis. Fractionation techniques have also been proven to be useful for the study of process-induced changes as well as for the classification of different feedstocks in specific clusters [17].

Considering the growing interest in the fraction containing insoluble-bound phenolics, it is important to choose the best solvent for the extraction of the soluble counterpart. Inefficient extraction of soluble phenolics may lead to overestimation of the insoluble-bound fraction. Therefore, investigation on the best solvent-assisted extraction conditions [172,173,174,175] has frequently been addressed. Besides chemical extractions, several studies have demonstrated that enzyme-assisted extraction may be a “green method” to recover phenolic compounds, including phenolic acids, monomeric flavonoids, proanthocyanidins, and anthocyanins [30,176,177]. In fact, recent findings have demonstrated that enzyme treatment should be considered for the development of nutraceuticals from plant by-products as the process changes the ratio of soluble/insoluble-bound phenolics; therefore, making them more physiologically bioaccessible, whereas insoluble-bound phenolics must be metabolized by the colonic fermentation before local biological action [30]. However, to evaluate the changes, a control for all steps (devoid of enzyme) should also be prepared to investigate pH and buffer effects. In fact, it is not difficult to find some studies evaluating the enzyme effect but failing to include a proper control. Therefore, it is not possible to ensure that the results actually reflect the action of the enzyme or arise from the solvent and/or pH effects. In this context, it is possible to find various studies supporting aqueous phenolic extraction [178,179,180], thus emphasizing the critical role of a proper control during enzyme-assisted extraction.

Different enzymes degrade distinct substrates [176]. Viscozyme has been found to be more effective than Pronase in releasing phenolic compounds from grape by-products [30]. The same study demonstrated that procyanidin dimer B, a major compound in this feedstock, was extracted with Viscozyme but not upon Pronase treatment. Even when the same enzyme is used, factors such as enzyme to substrate ratio, temperature, and incubation time may influence the extraction yield [176,181]. Regardless of the solvent chosen and/or the enzymatic treatment, especially for the extraction of the soluble phenolic fraction, the particle size of the feedstock also needs to be considered and properly reported. The antioxidant activity of cereal by-products has been found to be inversely correlated with the granulometry of the milling by-product sub-fraction [182]. Furthermore, conventional and non-conventional methods may be chosen for the extraction process, and the decision must be based on the feedstock, consumption of energy, and operation costs associated with the manufacturing facility [183]. Likewise, in terms of industrial application, the market value of the recovered compounds and final application, the cost of the solvents used and/or their removal (separation costs) as well as the cost associated with the use or developing of novel techonologies should be considered [184,185].

Ultrasound-assisted extraction resulted in a higher recovery of phenolics than the conventional solvent extraction [26]. Likewise, supercritical fluid extraction, especially under acidified conditions, rendered extracts rich in anthocyanins [27]. Furthermore, although Ferreres et al. [186] reported that temperature did not have any effect on the phenolic extraction of pitaya fruit by-products, another study conducted with winery by-products [8] demonstrated significant effects of temperature on the antioxidant activity of the recovered extract. These techniques have their importance, but they may affect the distribution of phenolic compounds (soluble versus insoluble-bound forms). Furthermore, even soluble conjugated phenolics may have their glycosidic moieties hydrolyzed during these processes [187]. Therefore, before using such techniques, a full characterization using classic chemical extraction procedures is needed to efficiently characterize all phenolics present, thus providing the basis for investigating their changes upon processing.

### 4.2. Estimation of Total Phenolic Content (TPC)

**Figure 4:**
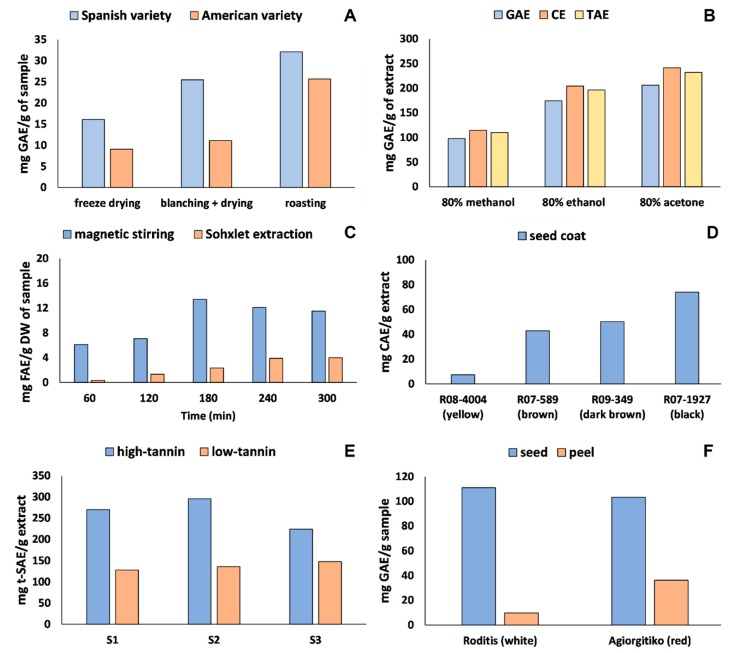
Total phenolics in selected plant food by-products. (**A**) Almond skin [191]; (**B**) hazelnut skin [192]; (**C**) rice husk [193]; (**D**) soybean coat [194]; (**E**) canola (high-tannin) and rapeseed (low-tannin) hull [195], S1, S2, and S3 are sample 1, 2, and 3, respectively; (**F**) grape seed and peel [196]. Abbreviations: GAE, gallic acid equivalents; CE, catechin equivalents; TAE, tannin acid equivalents; FAE, ferulic acid equivalents; CAE, chlorogenic acid equivalents; and SAE, sinapic acid equivalents.

The term quantification of total phenolics has long been used [188]. However, non-phenolic compounds may also react with Folin-Ciocalteu reagent [189]. According to Shinde et al. [190], the non-zero total phenolic content found in milk (devoid of phenolic extracts) may have resulted from milk proteins (e.g., tyrosine residues) and sugar components (oligosaccharide and glucose). Furthermore, different phenolic compounds have been found to react to varying degrees with this reagent. Therefore, expression of the results as a single number is necessarily arbitrary [189] and the trends among several samples, prepared under the same conditions, may be more important than comparing a single number. Therefore, the term quantification has recently been replaced by the estimation with respect to the evaluation of total phenolics [31]. Furthermore, the term estimation implies that in depth analysis (e.g., liquid chromatography–tandem mass spectrometry, LC-MS^n^) should be further carried out. Figure 4 details several examples from the literature which illustrate the complexity of making relevant interpretations based solely on TPC.

The study by Garrido et al. [191] (Figure 4A) demonstrated that total phenolics increased upon different heat processing operations. However, this may be misleading since a deeper evaluation of their data shows that some individual phenolics were not affected by the treatment (e.g., procyanidin trimer A) while some (e.g., eriodictyol-7-*O*-glucoside, kaempferol, isorhamnetin) were actually decreased after blanching and drying. It is important to note that this issue has not been fully tested. Therefore, those initiating their path into the chemistry of food phenolics should be careful in making quick interpretations.

The solvent system employed to recover phenolic compounds from hazelnut skin was studied by Contini et al. [192] (Figure 4B). According to these authors, regardless of the standards used (e.g., gallic acid, catechin, or tannic acid), 80% acetone rendered a higher extraction yield. It is of interest to note that catechin always gives higher values, followed by tannic acid and gallic acid. TPC provides an index or trend rather than an accurate quantification. Furthermore, as already mentioned, specific phenolic molecules exhibit distinct reactivity with Folin-Ciocalteu reagent, which stems from their different redox potential. Therefore, TPC are highly influenced by the standard used to calculate and report the final results.

Phenolics from rice husk (Figure 4C) were extracted using magnetic stirring or Soxhlet extraction over different time periods (60, 120, 180, 240, and 300 min) [193]. As for the method of extraction, magnetic stirring always rendered a higher yield. In contrast, increasing the extraction time was efficient only up to 180 min, after which, the TPC started to decrease, thus demonstrating that phenolic extraction is influenced by extration time.

The influence of the soybean seed coat was studied by Abutheraa et al. [194]. The darker the color, the higher was the TPC (Figure 4D). The tannin content (high versus low) also appears to have an influence on the TPC. Figure 4E shows that, irrespective of the sample, high-tannin canola always contained higher TPC [195]. Finally, one may think that by-products from red grape are the best source of phenolic compounds. However, while the peel of Agiorgitiko (red grape) presented higher TPC (Figure 4F), the opposite was noted for the seed of Roditis (white grape) [196]. Other colorimetric methods (e.g., total flavonoid and total proanthocyanidin) may also be used as screening tools. However, one should bear in mind the drawbacks of these methods.

The examples presented in this contribution and previous experiences demonstrate that comparing TPC results with those of the literature data may not be that informative. In contrast, comparing TPC results among several related samples (e.g., plant food by-products versus original material) [146] and/or fractions (e.g., soluble (free, esterified, etherified) versus insoluble-bound) [31] prepared by the same analyst and under the same conditions may serve as a screening method. In any case, if one decides to focus on TPC literature for compative purposes, a checklist could be helpful sto avoid misinterpretation. As illustrated in Figure 4A,D–F, different samples and/or varieties will likely show contrasting TPC values. Regardless of the test material, all steps involved in sample preparation (e.g., lipid, sugar and protein removal, particle size, solvent/enzymatic system, temperature, and time of extraction) and selection of phenolic standard must be checked. This critical checklist could be helpful to avoid overstatements that may influence the field of chemistry of phenolic compounds, especially in the emerging field of phenolics from plant processing by-products.

### 4.3. Identification and Quantification of Polyphenols

More than 8000 phenolic compounds have been reported in the literature, but just a few commercial standards are currently available, which demonstrates the critical role of hyphenated techniques such as liquid chromatography coupled to tandem mass spectrometry (LC–MS*^n^*) [197,198], matrix assisted laser desorption time of flight mass spectrometry (MALDI-TOF MS) [199] or other techniques [200,201,202]. Selected plant food by-products and screening of phenolics are summarized in Table 2.

## 5. Potential Health Benefits

### 5.1. Antioxidant Potential

Free radicals are related to lipid and protein oxidation, among others; which are detrimental to food and biological systems. Reactive oxygen species (ROS) are constantly generated via mitochondrial metabolism, which can worsen with unhealthy habits such as smoking [16]. ROS generated by immune cells may be beneficial to human health due to their role in preventing invasion of pathogens [2], however during homeostasis imbalance the body may not be able to neutralize ROS, which may lead to harmful effects. Overtraining by individuals engaged in intense exercise regimes is an example of homeostasis imbalance accompanied by oxidative stress [216]. A recent human trial with healthy adults under intense physical training demonstrated that phenolic compounds increase serum antioxidant status [217]. Some plant food by-products recently studied as a source of phenolic compounds and their proposed application areas are summarized in Table 3. The antioxidant potential of phenolic compounds from plant by-products has been substantiated by in vitro and in vivo studies [24,210]. The ability of polyphenols in scavenging free radicals may be explained by single electron transfer (SET) or hydrogen atom transfer (HAT) [218], which evidences the differences in their operative mechanisms. The number and position of hydroxyl groups in phenolic compounds are critical to their antioxidant potential. Therefore, polyphenols are, generally, more effective than monophenols. DPPH radical, ABTS (2,2′-azino-bis(3-ethylbenzothiazoline-6-sulphonic acid) diammonium salt) radical cation, and ORAC (oxygen radical absorbance capacity) are among the most commonly used methods for the first level screening of the antioxidant potential of natural products/compounds [219]. A recent report evaluated the mechanism of antioxidant action of some phenolic acids using ABTS radical cation and ORAC methods, the latter demonstrating the ability of an antioxidant to neutralize peroxyl radicals [220]. According to the authors [220], HOMO energy, rigidity (η) and Mulliken charge on the carbon atom in m-position to the phenolic hydroxyl were most significant descriptors of their antioxidant properties against peroxyl radical while electron transfer enthalpy from the phenolate ion was the most significant descriptor of the antioxidant capacity towards ABTS radical cation. The importance of ORAC method in the field of food bioactives and associated health benefits have also been reviewed [221], however, the shortcomings of this and other methods should also be considered [219]. The literature has also demonstrated the relevance of evaluating the antioxidant efficacy towards hydrogen peroxide, hydroxyl radicals, nitric oxide and peroxynitrous acid [2,222]. Furthermore, the ability of phenolic compounds towards superoxide anion and hypochlorous acid has been investigated [8,11].

### 5.2. Neutralization of Metal Ions

Metal ions also participate in redox reactions. Therefore, evaluating the capacity of phenolics in neutralizing them through chelation or reduction is also desirable. These methods may follow different mechanisms of action, therefore, using just one assay may not be sufficient for anticipating the actual effects using in vitro biological model systems as well as in vivo studies [16,17]. Furthermore, their results may differ according to the evaluation medium (e.g., solvent, buffer, and/or pH). Although some researchers still continue to ignore their use, the data collected by applying different methods may be useful. Similar to free radicals, metal ions are related not only to lipid oxidation [231], but also to protein oxidation [232]. It is well known that ferric ions and hydroxyl radicals are generated in the presence of hydrogen peroxide and ferrous ions through Fenton reaction (Haber-Weiss cycle). In this cycle, a set of dynamic redox reactions take place continually, at which ferrous ions are oxidized to the ferric form and the latter is again reduced to the ferrous form. It has been hypothesized that the ratio of ferric to ferrous ion is important for rapid initiation of lipid peroxidation through the Fenton reaction and the ratios of 1:1 to 7:1 (Fe^3+^/Fe^2+^) are optimum [231]. Therefore, an ideal antioxidant should not only be a good reducing agent but may also need to exhibit chelating capacity. Although the reducing power of food phenolics has been well substantiated, their chelating ability is not always easy to confirm. In fact, amongst 25 phenolics identified in berry seed by-products [24], protocatechuic acid was the only one showing a positive correlation with both reducing power (*r* = 0.8774, *p* = 0.002) and chelating capacity (*r* = 0.7430, *p* = 0.022), but a stronger correlation with reducing power was evident. The metal chelation of phenolics from grape by-products [233] was not correlated with any other assay, namely total phenolic content, DPPH radical scavenging and ORAC assay. Metal chelation takes place via complexation and, therefore, the chemical structures of polyphenols may have a higher influence on chelating ability than in the reducing power [19]. The latter study [19] demonstrated that free phenolics from pomegranate seeds showed about 2-fold higher ability in chelating ferrous ions than those released from their esterified form. Furthermore, a study by Andjelković [234] reported different binding constants for selected phenolic acids. Finally, according to the same research team, no complex formation was detected with compounds lacking a catechol or galloyl moiety.

The use of plant food by-products as a source of phenolic compounds has not yet been entirely taken advantage of by the industry. Among possible concerns is the microbiological safety, as mentioned earlier, but the presence of toxins produced by fungi and bacteria as well as potential presence of pesticide residues may contribute to the multitude of existing hurdles. Thus, studies involving humans may face more resistance, in need for prior investigations on their safety, collection of data in vitro, evaluation in cell lines, and in animal models. Regardless of the source, these processing by-products are rich in carbohydrates, fibre, protein, lipid, and minerals as well as a myriad of phytochemicals [33,163,224]. Besides the antioxidant potential, the most studied subject to date, some plant food by-products that have recently been considered as a source of phenolic compounds and remaining proposed application areas are summarized in the following sections.

### 5.3. Bioavailability of Phenolics

A wide range of potential health benefits through consumption of rich sources of phenolic compounds are addressed here with respect to their characterization and quantification in different source materials, including plant food by-products. However, less attention has been paid to research dealing with their bioavailability. The mechanism of action of food phenolics under physiological conditions remains unclear. In fact, the bioaccessibility of phenolic compounds in the small intestine or the metabolism of non-digestible phenolics upon fermentation in the colon may be pointed as one of the topics that remain to be clarified. These aspects play an important role in the bioaccessibility, which leads to the bioavailability and bioactivity of polyphenols and may be helpful to understand the discrepancies among different studies [235,236]. It has been accepted that extrapolations between in vitro and in vivo systems cannot be made [237]. These controversial results may also be found for ellagitannins [237]. Furthermore, high molecular weight phenolics may be broken down in the gastric juice [235]. Likewise, their degree of methylation and glycosylation may also be affected but, as far as we know, this point has scarcely been addressed.

The bioaccessibility and further bioavailability of phenolic compounds may explain the oxidative status of plasma as well as different tissues such as the liver, kidney, brain, and colon [238,239,240,241]; however, further confirmation is still necessary. The presence of phenolic metabolites in urine of rats treated with procyanidin A and B from apple and cranberry, respectively, may explain the mitigation of urinary tract infections in vivo [242]. Therefore, it is reasonable to suggest that the benefits of proanthocyanidins and ellagitannins stem, at least in part, from the action of their metabolites [237]. Although methylated, glucuronidated, and sulfated proanthocyanidins have been reported as metabolites of proanthocyanidin [235,236], the presence of unmodified proanthocyanidin in human plasma upon consumption of proanthocyanidin-rich foods has also been reported [243]. Likewise, ellagitanin metabolites such as urolithin A glucuronide, urolithin B glucuronide acid, urolithin-C glucuronide, urolithin-C methyl ether glucuronide, and dimethyl ellagic acid glucuronide have also been detected in human plasma following consumption of different sources of ellagitannins [244]. Therefore, studies focusing on the mechanism of action must be conducted not only with the native compounds but also with their biotransformed forms.

Within phenolic compounds, proanthocyanidins have been listed among the least absorbed [245]. Proanthocyanidins have been found in several by-products such as apple peel, blackberry, black raspberry, blueberry, litchi pericarp, as well as pomegranate peel [24]. The bioavailability of proanthocyanidins has been studied for a long time but until now, this subject is not entirely understood. While some authors have stated that proanthocyanidins with a degree of polymerization higher than four are not absorbed in the gut [236], others have reported that proanthocyanidins with an average degree of polymerization of six were absorbed by the epithelial cells [246]. The latter study showed that catechin as well as procyanidin dimer and trimer had similar permeability in colonic carcinoma (Caco-2) cells of human origin, which was close to that of mannitol, a known marker of paracellular transport, whereas proanthocyanidins with an average polymerization degree of six showed approximately 10 times lower permeability coefficients than the former molecules. In contrast, Ou et al. [247] demonstrated that procyanidin dimer, trimer and tetramers could cross Caco-2 cell monolayers but the ratio was decreased with higher degree of polymerization. Like proanthocyanidins (condensed tannins), ellagitannins (hydrolysable tannins) could be of high molecular weights and may not be readily bioavailable; therefore, low molecular weight phenolics such as ellagic acid must be released from their parent compounds to be absorbed and act as functional molecules.

In human intervention trials, Tomás-Barberán et al. [248] observed three different phenotypes for urolithin production upon ellagitannin and ellagic acid intake. According to these authors, “phenotype A” produced only urolithin A conjugates, whereas “phenotype B” produced isourolithin A and/or urolithin B in addition to urolithin A and no urolithins were detected in the third one, named “Phenotype 0.” The authors also highlighted that a higher percentage of phenotype B was observed in volunteers with chronic illnesses such as metabolic syndrome or colorectal cancer, which are associated with gut microbial imbalance. Therefore, especially for high-molecular weight phenolics and those linked to the cell wall of plant materials (insoluble-bound phenolics), the health status of the subjects should be carefully considered as it may affect the identities of the phenolic metabolites.

Human in vitro fecal fermentation studies demonstrated that even if proanthocyanidins (up to tetramers) as well as catechin and epicatechin were able to reach the colon, their presence would not be detected after colonic fermentation as the catabolites found were 5-(3′,4′-dihydroxyphenyl)-γ-valerolactone, (3,4-dihydroxyphenyl) acetic acid, protocatechuic acid, hydroxybenzoic acid, and salicylic acid. The same occurred with elagitannins as only gallic acid, pyrogallol, phlorogucinol, syringic acid, and protocatechuic acid were detected following in vitro fecal fermentation. Likewise, no anthocyanin was detected after fermentation and ferulic and sinapic acids present in oat and wheat bran were found in their hydrogenated forms as dihydroferulic acid and dihydrosinapic acid, respectively [249]. Therefore, although to a lesser extent compared to that of proanthocyanidins, ellagitannins, anthocyanins and monomeric flavonoids, phenolic acids may undergo biotransformation upon colonic fermentation after being released from their insoluble-bound form. However, at least with respect to ferulic and sinapic acids, their degree of hydroxylation may not change as demonstrated by Dall’Asta et al. [249]. Therefore, the biological effects of the parent compounds and phenolic metabolites may be similar.

### 5.4. Cardiovascular Diseases

Prevention of atherosclerosis and associated cardiovascular diseases (CVD) has been suggested among the potential health benefits of food phenolics. Low-density lipoprotein-cholesterol (LDL-C) levels have been found to be significant predictors of death from cardiovascular and coronary heart disease in men with and without preexisting cardiovascular disease in a ten-year mortality study [250]. The same study also concluded that low levels of high-density lipoprotein cholesterol were significant predictors of death from CVD. According to Martín-Carrón et al. [251], commercial dietary fiber products rich in polyphenols can be obtained from red and white whole grape pomace produced after wine or grape juice production, as well as from white and red skins and seeds. The study conducted by these authors reported the reduction in LDL-C concentrations due to the consumption of a diet supplemented with a dietary fiber and polyphenols rich product in hypercholesterolemic rats.

Winemaking by-products (100 mg/kg/d) also showed biological activity by decreasing the levels of VLDL-cholesterol and triacylglycerols in Wistar rats [15]. In addition, Aviram et al. [252] evaluated the antiatherogenic properties and mechanisms of action of different pomegranate fruit parts (peels arils, seeds, and flowers) and the atherosclerotic lesion area was significantly decreased by up to 70%. The presence of oxidized LDL-C is also involved as an early event in the pathogenesis of atherosclerosis, a condition where plaque inside the arteries may impair the blood flow and increase the risk of coronary heart disease. Development of atheromatous plaques takes place due to the uptake of oxidized LDL-C, via scavenger receptors, thus leading to cholesterol accumulation and foam cell formation [21,223,253]. Phenolic antioxidants act as chain breakers through inhibition of lipid peroxidation and may also inhibit oxidation of protein, thus potentially preventing LDL-C via multiple mechanisms [164]. Metal ion-catalyzed oxidation of proteins and lipids have several consequences. Therefore, methods such as the cupric ion induced human LDL-C peroxidation have been useful in demonstrating the potential benefits of phenolic compounds in reducing the risk of CVD. This topic was discussed in an editorial, highlighting its importance for prospection of new sources of phenolic compounds to reduce and/or prevent CVD [254].

The lowest inhibition of lipid peroxyl radical species of gallic acid as compared with epigallocatechin gallate lends support to the importance of the lipophilicity of phenolic compounds [255]. In addition, lipophilised epigallocatechin gallate ester derivatives were more effective than epigallocatechin against cupric-induced LDL-C peroxidation [256], which was in good agreement with a recent study [257]. Therefore, inhibition of protein oxidation of LDL-C may also be contemplated, and these studies allow to suggest that oxidation of the lipid fraction may be more important compared to the protein components. The chelation capacity of phenolic compounds towards copper ions may also be involved. Besides pure compounds and their lipophylized derivatives, phenolics from several by-products from legumes, oilseeds, cereals and fruits have been investigated [16,17,19,21,24,207,223]. The IC_50_ of free, esterified and insoluble-bound phenolic extracts from camelina and sophia seed meals was in the range of 20–30 µg/mL [223], whereas the corresponding values found for chia meal were between 20 and 70 µg/mL.

Studies on pomegranate by-products demonstrated that some phenolic fractions could inhibit LDL-C oxidation in vitro [19,21], however, the inhibition was not correlated with the total phenolic content. In a previous study, de Camargo et al. [16] demonstrated that only eight, out of 18 phenolics quantified in grape by-products were correlated with LDL-C inhibition, which was confirmed by the study conducted by Ayoub et al. [24]. These studies support the role of the chemical structure on the antioxidant capacity in a complex system containing both lipid and protein fractions and oxidants such as metal ions as well as ROS and NOS. The great potential in improving the cardiometabolic profile of food phenolics in animal models and in humans is well substantiated, however, considering the emerging market of plant food by-products as potential sources of phenolic bioactives, more research in humans is deemed necessary.

### 5.5. Phenolics as Adjuvants in Cancer Prevention and Treatment

Cancer has been listed by the International Agency for Research on Cancer (IARC), among the worldwide leading diseases. Lung, liver, colorectal, stomach, and female breast have been pointed as the most common causes of cancer death. Carcinogens may be of chemical (tobacco smoke and mycotoxins) and physical (e.g., ultraviolet and ionizing radiation) nature. Furthermore, infections from certain viruses, bacteria, or parasites are examples of biological carcinogens. Several mechanisms that account for the anticarcinogenic actions of phenolic compounds and culminate in apoptosis and/or cell cycle arrest have already been summarized [258,259]. However, DNA-damage signaling and repair have been highlighted as crucial pathways to the etiology of most, if not all, human cancers [260]. In this sense, DNA strand breakage may lead to mutagenesis and affect its replication and transcription, which is among the causes of cancer initiation [24,197].

Mycotoxins have been listed among the chemical carcinogens and, in fact, some studies have reported mycotoxin-DNA damage in vitro and in vivo [261,262]. The presence of these potential carcinogens has been reported in several food and processing by-products [148,263,264,265]. Furthermore, the observed DNA damage has been linked to oxidative stress. As mentioned in a previous report, avoiding consumption of these products may or may not be a realistic option [164]. Furthermore, to address this question, the protective effect of phenolic compounds to overcome deleterious effects due to exposure to mycotoxins has been a target of studies.

Long et al. [266] reported the protective effect of grapeseed proanthocyanidin extract on oxidative damage induced by zearalenone in Kunming mice and suggested that the mechanism could be related to the activation of the Nrf2/ARE signaling pathway. Furthermore, proanthocyanidins were found to protect against acute zearalenone-induced testicular oxidative damage in male mice [267]. Zearalenone metabolites (α- and β-zearalenol) have been studied by Ben Salem et al. [268]. According to these authors, quercetin was able to protect cells against α- and β-zearalenol-induced endoplasmic reticulum stress and apoptosis. In another study, quercetin was found to prevent endoplasmic reticulum stress and reduce zearalenone-induced apoptosis in HCT116 and HEK293 cells [269]. Therefore, considering the literature, it is possible to suggest that mycotoxin-induced changes may be, at least in part, faced as one type of oxidative stress imbalances, and as rich sources of phenolic compounds, plant food by-products may be useful to overcome mycotoxin-related issues.

Peroxyl and hydroxyl radical induced supercoiled DNA strand scissions have been useful to demonstrate potential benefits of phenolic compounds in reducing the potential risk of certain types of cancer. Phenolics from several plant food by-products inhibited ROS-induced DNA damage in vitro [17,19,21,24,171,207,229]. Furthermore, some studies in cell models and in vivo have substantiated the anticancer potential of phenolics from agro-industrial residues [270,271]. Free and soluble-conjugate phenolics are, at least partially, bioacessible and could reach the plasma and different tissues, which may explain the preventive effect of food phenolics towards certain types of cancer. On the other hand, colorectal and stomach cancer prevention may not necessarily be related to the bioaccesibility of polyphenols. As mentioned before, colorectal cancer has been listed among the most common causes of cancer death. Pan et al. [258] summarized the molecular mechanisms for chemoprevention of colorectal cancer by natural dietary compounds, including polyphenols. Another study demonstrated that phenolics from the outermost fraction of barley showed a high level of antiproliferative activity toward inhibition of Caco-2 human colorectal adenocarcinoma cells [45]. Insoluble-bound phenolics from grape by-products have been found to be the major fraction compared to the soluble counterpart [16,30]. Additionally, it has been accepted that phenolic compounds in the insoluble-bound form are not readily bioacessible. However, by being metabolized by human colonic microbiota, phenolics present in the insoluble-bound may be released in the colon and prevent colorectal cancer.

Besides the preventive effect of long-term consumption of rich sources of phenolic compounds, these natural compounds may also act as adjuvants during cancer treatment. Surgery, chemotherapy, radiation, or their combination, are the most common treatments for cancer [164]. Several side effects such as nephrotoxicity, neurotoxicity, hepatotoxicity, cardiotoxicity, as well as gastrointestinal and pulmonary toxicity have been reported for several drugs used in the treatment of cancer [272]. Furthermore, the oxidative stress-based hypothesis involving production of ROS due to the use of anticancer drugs has gained acceptance [273].

Doxorubicin, an anthracycline, generates hydrogen peroxide, hydroxyl, and superoxide radicals as a result of oxidative metabolism. The lower oxidative stress, compared to the control, in anthracycline treated rats was attributed to catechin [274]. The ability of irradiation in impairing the growth and multiplication of cancer cells is related to DNA damage probably due to oxidation thus generating ROS. Although normal cells may also be affected by the treatment, they have a greater ability in repairing themselves and overcome exposure to radiation [164].

The ability of human lymphocytes in vitro in rejoining from X-ray-induced DNA double-strand break has been found to be dependent on the age of subjects, and older ones showed lesser ability in overcoming DNA damage than that found in DNA from younger blood donors [275,276]. According to Singh et al. [275], DNA repair from X-ray induced damage was more difficult in older individuals. Due to the crucial role of phenolic compounds towards ROS-induced DNA strand breakage, evaluation of their protective effect against DNA damage may also be used to anticipate their potential in alleviating drug- and radiation-induced effects during cancer treatment [277].

### 5.6. Type 2 Diabetes and Obesity

At a molecular level, the ability of phenolic compounds in inactivating digestive enzymes has been shown to be a good option in several pre-clinical studies [6,278]. Carbohydrate- (α-amylase and α-glucosidase) and lipase-hydrolysing enzymes present in the small intestinal brush border participate in the breakdown of complex carbohydrates and triacylglycerols and enable their absorption. Inhibitors of carbohydrate-hydrolysing enzymes are able to retard the liberation of d-glucose from dietary complex carbohydrates thus delaying glucose absorption which, in turn, may reduce postprandial plasma glucose levels and suppress postprandial hyperglycemia [279].

Although anti-hyperglycemic products are available in the market, their use may result in several side effects. Additionally, anti-hyperglycemic drugs are provided by the government free of charge in some countries like Brazil, thus becoming a national economic burden [30]. Phenolics bearing digestive enzyme inhibitory activity and their respective inhibition capacity are shown in Table 4.

According to the literature [31], the enzyme inhibition capacity of polyphenols may be explained by their complexation with proteins through hydrogen-bonds or addition of nucleophiles to oxidized quinones [282]. Therefore, the general understanding that oxidized phenolic compounds do not serve as bioactives may be misleading. In fact, oxidation of polyphenols is an intermediary step for further nucleophilic reaction with several enzymes. Protein-binding is dependent on several factors such the size, length, and flexibility of phenolic compounds. Furthermore, the stereospecificity of polyphenols and proteins is also important [283]. It has been hypothesized that larger molecules (e.g., proanthocyanidins) are more likely to bind with proteins as compared to low molecular weight phenolics [284]. However, whereas polymeric proanthocyanidins have shown higher inhibitory effect towards α-amylase, the opposite was found against α-glucosidase [285], for which oligomeric proanthocyanidins were actually more effective. Therefore, generalizations are not as simple as one would expect.

Acarbose, an oral anti-diabetic drug, was found to be a competitive inhibitor for α-glucosidase and mixed noncompetitive inhibitors for α-amylase [286]. Inhibition studies demonstrated that proanthocyanidins were a mixed noncompetitive inhibitor against α-amylase but a competitive inhibitor against α-glucosidase [180]. Inhibitory effects of phenolic compounds towards enzyme activity are not as linear as found against DPPH radical and ABTS radical cation. Therefore, the enzymatic inhibition is frequently reported as a percentage of inhibition or in terms of IC_50_, the concentration necessary to inhibit enzymatic activity by 50%. A recent study [31] demonstrated that free phenolics of peanut skin exhibited a lower IC_50_ than that of acarbose [6]. The IC_50_ for several phenolics from different sources has been summarized by Kumar et al. [279].

Environmental conditions have been accepted as a critical factor influencing the phenolic profile of grapes and hence, their processing by-products. However, Kadouh et al. [287] evaluated grape pomaces from six grape varieties grown in the same vineyard and suggested that the greatest inhibitory effect of Tinta Cão grape by-products towards α-glucosidase stems from varietal effects rather than agronomic conditions. The correlation of total phenolics with antioxidant potential and reducing power of several plant by-products is well established [16,24], but the correlation between α-glucosidase with total phenolics has also been reported [30,31,287], thus indicating a dose-dependent response.

Recently, metabolomic analysis enabled the identification of several potential anti-α-amylase agents, namely epigallocatechin gallate, herbacetin-3-*O*-d-glucopyranosyl-7-*O*-l-rhamnoside, kaempferol 3-xylosyl-(1→6)-glucoside, berbacetin-8-*O*-d-glucopyranoside, tricin 7-*O*-β-d-glucopyranoside, kaempferol 3-*O*-glucoside, tricin 5-*O*-β-d-glucopyranoside, herbacetin-7-*O*-rhamnoside, kaempferol and tricin [288]. In contrast, commercial standards, namely catechin, resveratrol, delphinidin chloride, cyanidin chloride, malvidin-diglucoside, malvin chloride, malvidin chloride, cyanidin-diglucoside, procyanidins B1 and B2, epicatechin gallate, kaempferol, myricetin, quercetin hydrate, quercetin 3-*O*-glucoside, and phenolic acids (gallic, caffeic, *p*-coumaric, and ferulic acids) were tested against rat intestinal α-glucosidases, but no inhibition was found [287]. The authors therefore concluded that unidentified bioactive components from their starting material could be responsible for inhibiting the enzyme. The same research team [6] conducted a subsequent bioactivity-guided isolation and purification of α-glucosidase inhibitor from Tinta Cão grape pomace and confirmed the inhibitory ability of 6-*O*-*p*-*trans*-coumaroyl-d-glucopyranoside, which was not identified in their previous study [287].

Masumoto et al. [289] demonstrated that mice fed a high-fat/high-sucrose diet administered along with non-absorbable apple procyanidins showed lower levels of endogenous metabolites associated with insulin resistance as compared with the control group. Several proanthocyanidin-rich plant food by-products such as peanut skin, persimmon peels, and grape by-products, among others [30,31,285] have been pointed as good options to inactivate digestive enzymes (e.g., α-amylase and α-glucosidase). In addition, rich sources of hydrolysable tannins, anthocyanins, phenolic acids of meal from dry-blanched peanuts and pomegranate by-products also exhibited inhibitory properties [19,31]. Finally, the synergistic effect of cyanidin-3-galactoside with acarbose has also been reported [290], suggesting that anthocyanins and other potential phenolic compounds may improve the effects of acarbose for treatment of diabetes, thus encouraging their combined use.

It is already common sense among health professionals that obesity is associated with a higher risk of developing several chronic ailments (e.g., type 2 diabetes, cardiovascular diseases, and certain types of cancer). Therefore, body weight management may decrease the risk of many diseases and their complications. Orlistat, a conventional anti-obesity drug, has been used as a positive control in many studies [281,291]. The mechanism behind the interaction of food phenolics with lipase may be similar to α-amylase and α-glucosidase, however, phenolic-enzyme specificity may differ, as discussed earlier.

In this context, phenolics from pomegranate by-products [19] exhibited, generally, a higher inhibitory effect towards α-glucosidase than amongst lipase. Proanthocyanidin-rich fractions from pecan shell [292] also showed inhibition towards α-amylase and pancreatic lipase, which was dependent on the degree of proanthocyanidin polymerization. Furthermore, the influence of phenolic content has not been found to be as drastic as it was on antioxidant activity and reducing power [31]. In fact, up to 28-fold higher total phenolic content was found in the phenolic-rich fractions from peanut skin as compared with the fractions obtained from peanut meal from dry-blanched samples. However, 1.8-(α-glucosidase) and 2.2-fold (lipase) higher inhibition was found in the most active samples. These results suggest that the number of hydroxyl groups in phenolic compounds to deactivate digestive enzymes is not as important as they are towards reactive oxygen species and metal ions.

Myricitrin and quercitrin have shown dose-dependent lipase inhibitory effects, but the first one had a stronger inhibitory activity [7]. According to Zhang et al. [7], molecular docking analysis showed that myricitrin bound more tightly than quercitrin to the lipase with a greater number and shorter distance of hydrogen bonds, which supported their experimental results. As for the structure-activity, sugar-removed proanthocyanidin extracts showed higher anti-obesity effects in rat models [291] than that of glycosylated proanthocyanidin extracts.

Polyphenols modulate energetic metabolism, glucose uptake, absorption of cholesterol, and production of apolipoproteins. Furthermore, phenolic compounds also affect hormones related to satiety by downregulation of grelin and upregulation of leptin [293]. For example, proanthocyanidin extracts exerted their anti-obesity effects by upregulating the expression of SIRT1, thus inducing the deacetylation of PPAR-γ and downregulating the expression of C/EBP-α, as well as upregulating the expression of BMP4 to boost the levels of brown fat [291].

Besides inhibiting lipase activity in vitro [278], this study demonstrated that proanthocyanidins also reduced the accumulation of total triacylglycerols and cholesterol induced by oleic acid in HepG2 cells. Although another study [31] reported a low potential of proanthocyanidin rich-fractions from peanut skin compared to that of orlistat, Zhang et al. [278] supported the hypolipidemic property of these compounds. These authors reported that proanthocyanidins significantly increased the phosphorylation of AMP-activated protein kinase (AMPK) and thus reduced triacylglycerols and sterols biosynthesis by inhibiting its downstream proteins, such as acetyl-CoA carboxylase, 3-hydroxy-3-methylglutaryl-CoA reductase and sterol regulatory element-binding protein. They also stated that proanthocyanidins regulated cellular glucose metabolism by promoting glucose consumption in HepG2 cells, thus lending support to their use in preventing and/or managing hyperglycemic diseases.

*Myrciaria jaboticaba* peel containing phenolics such as cyanidin and ellagic acid prevented fat weight gain and decreased peripheral insulin resistance in animal models [294]. In another study, freeze-dried jaboticaba peel was found to decrease saturated fatty acids of rats fed high-fat diets. Fecal triacylglycerols also increased in high-fat-diet groups (obese rats) given freeze-dried jaboticaba peel, which showed a dose-dependent response to anthocyanins intake, thus suggesting the role of anthocyanins in jaboticaba by-products in lowering the absorption of triacylglycerols in vivo [238]. In humans, the intake of *M. jaboticaba* peel decreased glucose and insulin levels, which indicates important clinical effects, such as improvement of insulin sensitivity [295].

The correlation between oxidative stress and obesity [296] has been associated with mitochondrial and hepatic dysfunction and endoplasmic reticulum stress [297]. As mentioned before, polyphenols are classically known to reduce oxidative stress [297] by scavenging free radicals via modulation of redox-sensitive enzymes and NRF2, among other mechanisms [297,298]. In this way, ingestion of polyphenols has been associated with the prevention and control of oxidative stress in obesity [238].

Gut microbiota can biotransform phenolic compounds, producing aglycones and other derivatives [299] and, on the other hand, polyphenols can also play a role by modulating gut microbiota in such a prebiotic-like effect [300]. Phenolic compounds are metabolized in the gut and modulate colonic microbiota [300]. Gut microbiota transforms nutrients and other dietary components into several metabolites modulating the human immune system and metabolic responses. In this way, as literature more and more correlates obesity and comorbidities with gut microbiota [301], understanding how polyphenols can modulate intestinal bacteria composition is crucial to better understand the role of polyphenols in health promotion.

In summary, randomized controlled trials have shown that diets rich in polyphenols are associated with reduced obesity parameters [302]. Evidence from studies in vitro, using animal models and clinical trials [31,294,295], show that several mechanisms could be involved, like modulation of digestive enzymes, gut microbiota and finally energy metabolism. Furthermore, polyphenols also modulate low grade inflammation [303], and this topic will be addressed below.

### 5.7. Anti-Inflammatory Effects

Inflammation is a host defense response against an invading agent which involves the participation of immune system cells, such as neutrophils, macrophages and lymphocytes [304,305]. Despite beneficial effects, the occurrence of an inappropriate inflammatory process may commonly trigger the onset of inflammatory disorders, for instance, rheumatoid arthritis and diabetes mellitus [306,307]. The daily intake of rich sources of phenolic compounds has been directly associated with disease prevention, since these substances produce biological effects in the body, particularly due to their antioxidant and anti-inflammatory properties [308]. Studies have shown that such a broad class of bioactive compounds is capable of modifying different pathways during the inflammatory process, by acting through enzymatic inhibition, antagonism of extracellular and intracellular receptors, modulation of cell signaling pathways, and synthesis of proinflammatory cytokines [308,309,310,311,312].

Recent research has shown the anti-inflammatory potential of several plant food by-products using in vitro and in vivo experimental models and further isolated and/or characterized the major bioactive substances present therein [8,313,314,315]. Among the by-products, the pomace and seeds of different types of grapes have attracted attention for their anti-inflammatory potential and phenolic composition. Denny et al. [209] detected the presence of proanthocyanidins, flavan-3-ol monomers and anthocyanins in the pomace of Petit verdot grapes. The authors further reported that administration of the extract and fractions from Petit verdot grape pomace reduced inflammation by inhibiting tumor necrosis factor α (TNF-α) and interleukin-1 beta (IL-1β) in mice.

Proanthocyanidins have also been identified in grapeseed and have shown to be responsible for modulating the experimental inflammatory response. A study by Chu et al. [225] demonstrated that a grape seed proanthocyanidins extract (90% polyphenols, which consists of a combination of ingredients with more than 85% oligomeric proanthocyanidins (OPCs) and more than 7% (+)-catechin and (−)-epicatechin) was able to suppress the release and expression of inflammatory cytokines by lipopolysaccharide-stimulated macrophages (LPS). The authors reported that the mechanism of action of the extract is related to (i) inhibition of phosphorylation of the mitogen-activated protein kinase (MAPK) and (ii) inhibition of activation of the nuclear factor kappa B (NF-κB). The effects of the grape seed proanthocyanidins extract were confirmed by Park et al. [226] using a model of inflammatory disease. These authors showed that the extract containing 98.5% proanthocyanidins, was effective in reducing bone loss associated with arthritis-induced inflammation. In another study [227], administration of the grape seed proanthocyanidin extract led to an improvement in the renal lesion of type 2 diabetic rats, further confirming its beneficial effects.

Tatsuno et al. [213] isolated several proanthocyanins and tested the biological activity of the peanut skin extract as well as of the isolated proanthocyanidins against the production of inflammatory cytokines in a human monocytic THP-1 culture. The results indicated that the peanut skin extract decreased the production of TNF-α and interleukin 6 (IL-6) by THP-1 cells in response to LPS. As for the biological activity of the isolated compounds, proanthocyanidin dimers and trimers were more potent compared to monomers or tetramers. Procyanidin B2 has been identified in the peel of two different varieties of avocado, while Procyanidin B1 was present only in the seed. Furthermore, epicatechin, another major compound, was present in both peel and seeds [11]. According to the authors, these compounds were responsible for the inhibitory activity of the phenolic extracts obtained from these by-products towards the release of TNF-α cytokine and nitric oxide by LPS-stimulated macrophages. In line with that, Denny et al. [228] identified the compounds epicatechin, quercetin, myricetin, isovanilic and gallic acid in the pomace extract of *Psidium guajava* L. The pomace extract reduced paw edema and peritonitis in carrageenan-challenged mice.

Different studies have provided scientific evidence that pomegranate by-products are rich in phenolic compounds and possess significant anti-inflammatory activity [19,21,316,317]. Park et al. [214] examined the effects of pomegranate peel extract on stimulated THP-1 cells. The extract was found to have a suppressive effect on the production of free radicals, the expression of TNF-α, IL-1β, monocyte chemoattractant protein-1 (MCP-1) and the intercellular adhesion molecule 1 (ICAM-1) and led to reduced monocyte adhesion to endothelial cells. Such inhibitory activity on monocyte adhesion to endothelial cells was also observed for the compounds punicalagin and ellagic acid, which can be found in the pomegranate peel [214].

**Figure 5:**
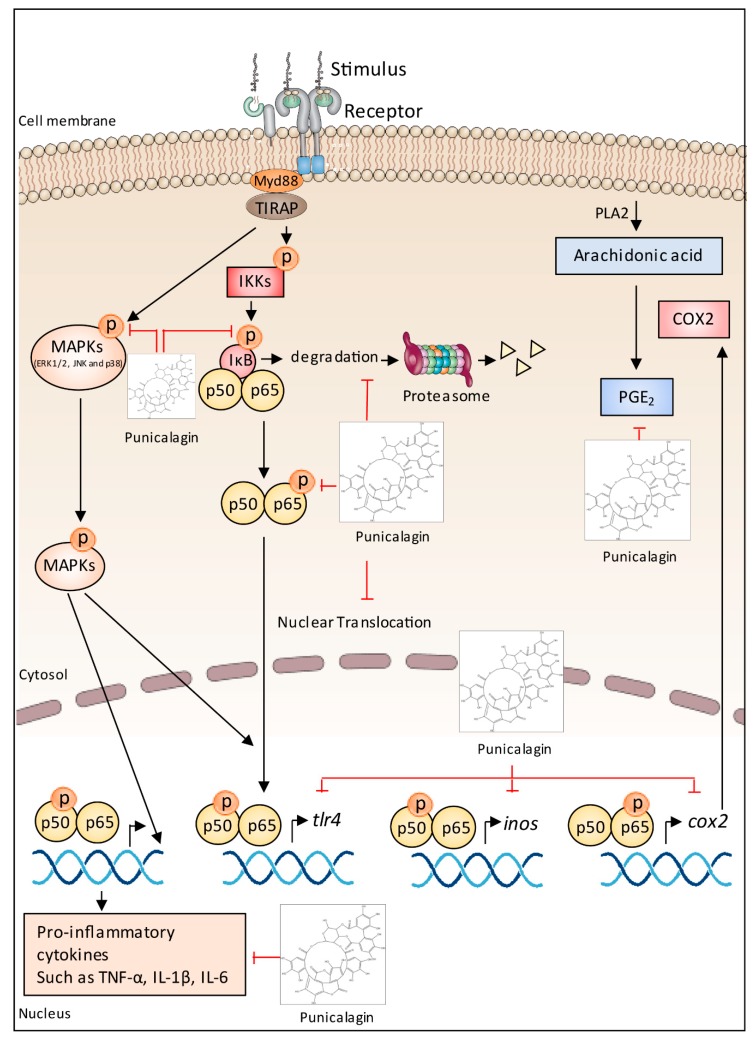
Anti-inflammatory mechanisms of punicalagin. Triangles represent degraded IkB.

Punicalagin shows anti-inflammatory activity and is among the major compounds of pomegranate [316]. A study carried out by BenSaad et al. [318] showed that punicalagin was able to inhibit the production of nitric oxide, prostaglandin E2 (PGE2) and IL-6 in stimulated RAW 264.7 macrophages. The molecular mechanisms of action of punicalagin on cell signaling (Figure 5) were elucidated by Xu et al. [319] and Kim et al. [320].

According to Xu et al. [319], the inhibitory activity of punicalagin on the release of inflammatory cytokines occurs through the reduction of NF-κB activation, as well as through reduction of phosphorylation of the MAPK c-Jun N-terminal kinase (JNK), extracellular signal-regulated kinases (ERK) 1/2 and p38 mitogen-activated protein kinases (p38 MAPK). Both effects were shown to be related to the inhibition of overexpression of the Toll-like receptor mRNA 4. In addition, Kim et al. [320] found that punicalagin suppresses the activation of NF-κB by preventing degradation of IκB as well as the translocation of p50 and p65 to the cell nucleus. The authors further showed that punicalagin inhibited the expression of iNOS and COX2.

**Figure 6:**
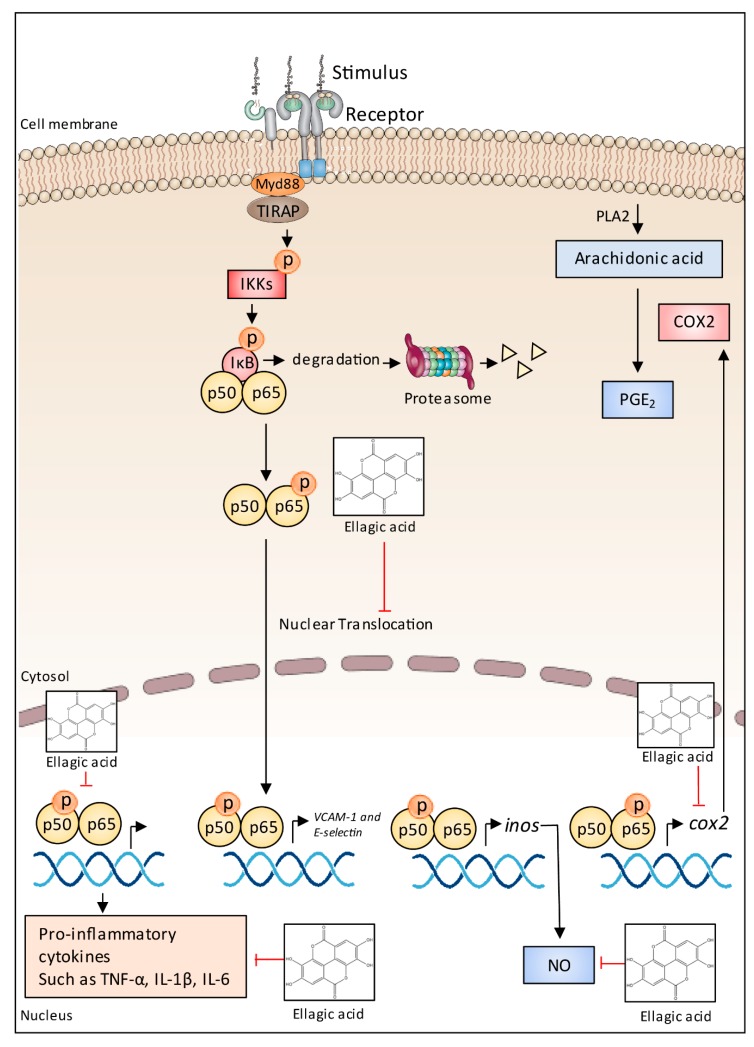
Anti-inflammatory mechanisms of ellagic acid. Triangles represent degraded IkB.

Ellagic acid, also one of the most prominent compounds in pomegranate by-products, had its anti-inflammatory activity evaluated and the mechanisms of action mechanism of action elucidated (Figure 6) [321,322,323].

BenSaad et al. [318] also showed that ellagic acid is able to reduce nitric oxide, PGE2 and IL-6 production in stimulated RAW 264.7 macrophages. El-Shitany et al. [324] reported that administration of ellagic acid reduced acute inflammation in mice. According to these authors, the mechanism of action is, at least in part, related to reduction of nitric oxide, IL-1β, TNF-α, COX-2 and NF-kB. As for the chronic inflammation, Allan et al. [325] demonstrated administration of ellagic acid attenuated arthritis development by reducing proinflammatory cytokines. According to Yu et al. [326] ellagic acid reduced the expression of VCAM-1 and E-selectin as well as the adhesion of monocytes to endothelial cells. The reduction of VCAM-1 and E-selectin expression was explained by the inhibition of nuclear translocation of p65 and p50.

Polymethoxyflavones are a subclass of flavonoids which have also attracted interest because of their potential to modulate the inflammatory process by blocking the expression of endothelial cell adhesion molecules and inhibiting the release of inflammatory cytokines [327]. These bioactive substances were found in sweet orange peel, which adds economic value to its by-product [212]. In addition to sweet orange, the anti-inflammatory activity of berries and their by-products have also been investigated [328,329,330]. A study carried out by Park et al. [205] found that the tannin fraction obtained from black raspberry seeds was able to reduce the levels of nitric oxide in a culture of stimulated macrophages. The chemical composition of the tannin fraction mainly showed the presence of ellagitannins, which are substances with diverse biological effects in the organism, including anti-inflammatory properties.

## 6. Conclusions

Plant food by-products have attracted much attention due to their potential as a source of bioactive compounds. Phenolic compounds are of special interest due to their preventive action against cardiovascular disease and certain types of cancer, which have been linked to the antioxidant activity, reducing power, and chelation capacity of these phytochemicals. Increasing interest of their action in the management of metabolic disorders such as diabetes and obesity has also been found. In addition, polyphenols may render anti-inflammatory effects. For these reasons, their application in the area of functional foods and nutraceuticals has been recommended. However, some hurdles and challenges should be addressed, as discussed in this review which also briefly summarized some of them, which included the safety, characterization, and evaluation of potential health benefits. Genetic control of phenolic biosynthesis is complex and involves a matrix of overlapping regulatory signals during plant development. For some of the key compounds, such as flavonoids, there is now a very good understanding of the nature of those signals and how the signal transduction pathway connects to the activation of phenolic biosynthetic genes. As for the safety, more attention should be paid to the microbiological and toxicological aspects of the starting material and final product. Identification should take into account the fraction containing soluble phenolics, and the insoluble-bound fraction must be included. Literature on the use of alkaline versus enzymatic extraction is scarce. Identification of phenolics still suffers from lack of commercial standards, therefore the use of HPLC itself is not the best tool for such a purpose, thus the use of hyphenated techniques (e.g., LC-MS*^n^*) is deemed necessary. Consequently, development of a functional ingredient or nutraceutical should consider all these aspects.

## Figures and Tables

**Table 1:** Role of phenolic compounds in plants.

Compounds	Function	References
**Monomerics**		
Phenolic acids	Protection against infection of microbes, improvement of nutrient uptake, protection against insect depredation, signaling molecules in plant-microbes symbioses, involvement in plant allelopathy.	[46,47,48,49,50,51,52]
Flavonoids	Attract pollinators and seed dispersers, protection against oxidative stresses derived from UV, high light, and low temperatures, preventing photoinhibition and photobleaching, regulation of auxin transport, modulation of flower color, protection from high intensity light and UV, protection against DNA damage, involvement in plant allelopathy, antimicrobial activity, regulation of *Rhizobium* nodulation genes, protection against depredation by herbivores.	[53,54,55,56,57,58,59,60,61,62,63]
**Dimerics, oligomerics, and polymerics**		
Proanthocyanidins	Protection against depredation by invertebrates and vertebrates, scavenging of reactive oxygen species, protection against microbes infection.	[64,65,66]
Hydrosable tannins	Protection against wounds and depredation by microbes and herbivores.	[67,68,69,70]
**Cell wall materials**		
Lignins	Lodging resistance, involvement in plant fertility, mechanical barrier in seeds, biotic and abiotic stress resistance, involvement in plant growth and development.	[71,72,73,74,75]
Lignans	Scavenging of reactive oxygen species and antimicrobial activity, protection against insect depredation, involvement in plant allelopathy, phytohormone-like property.	[76,77,78,79]

**Table 2:** Selected plant food by-products and phenolic screening.

Feedstock	Product Fraction	Phenolic Compounds	Method *	Ref.
Almond	Skin	Proanthocyanidins	HPLC-MS	[203]
Apple	Peel	Phenolic acids and monomeric flavonoids	UPLC-MS	[151]
Avocado	Peel and seed	Phenolic acids and flavonoids	HPLC-MS	[11]
Barley	Outermost milling fraction	Phenolic acids	HPLC	[204]
Blackberry	Seed meal	Phenolic acids, monomeric flavonoids, proanthocyanidins, and anthocyanins	HPLC-MS	[24]
Black raspberry	Seed meal	Phenolic acids, monomeric flavonoids, proanthocyanidins, and anthocyanins	HPLC-MS	[24]
Black raspberry	Seed	Ellagitannins and proanthocyanidins	HPLC-MS	[205]
Blueberry	Wine pomace	Anthocyanins	HPLC-MS	[26]
Blueberry	Seed meal	Phenolic acids, monomeric flavonoids, proanthocyanidins, and anthocyanins	HPLC-MS	[24]
Brazil nut	Skin	Phenolic acids, monomeric flavonoids, and proanthocyanidins	HPLC-MS	[80]
Camelina	Seed meal	Phenolic acids, monomeric flavonoids, and proanthocyanidins	HPLC-MS	[206]
Chia	Seed meal	Phenolic acids, monomeric flavonoids and proanthocyanidins	HPLC-MS	[207]
*Citrus reticulata*	*Chempi* (aged peel)	5-demethylated polymethoxyflavones	HPLC	[208]
Grape	Pomace	Phenolic acids, monomeric flavonoids, proanthocyanidins, and anthocyanins	HPLC-MS	[209,210]
Grape	Pomace and rachi	Phenolic acids, monomeric flavonoids, proanthocyanidins, and anthocyanins	HPLC	[8]
Grape	Winemaking and grape juice by-products	Phenolic acids, monomeric flavonoids, and proanthocyanidins	HPLC-MS	[16]
Mango	Residual pulp	Phenolic acids and monomeric flavonoids	HPLC	[38]
Millet	Hull	Phenolic acids	HPLC-MS	[171]
Onion	Skin	Monomeric flavonoids	HPLC-MS	[211]
Orange	Peel	Flavonoids	HPLC-MS	[22]
Orange	Peel	Polymethoxyflavones	HPLC-MS	[212]
Passion fruit	Peel, albedo and seed	Phenolic acids and monomeric flavonoids	HPLC	[38]
Peanuts	Skin and meal	Phenolic acids, monomeric flavonoids, and proanthocyanidins	HPLC-MS	[12,31]
Peanuts	Skin	Phenolic acids, monomeric flavonoids, and proanthocyanidins	HPLC-MS	[17,144]
Peanuts	Skin	Proanthocyanidins	HPLC	[18,213]
Pineapple	Peel, and residual pulp	Phenolic acids and monomeric flavonoids	HPLC	[38]
Pomegranate	Peel and seed	Phenolic acids, monomeric flavonoids, anthocyanins, proanthocyanidins, and ellagitannins	HPLC-MS	[19,21]
Pomegranate	Peel	Punicalagin and ellagic acid	HPLC	[214]
Soybean	Okara	Isoflavones	UPLC	[215]
Soybean	Seed coat	Phenolic acids and flavonoids	HPLC-MS	[40]
Sophia	Seed meal	Phenolic acids, monomeric flavonoids, and proanthocyanidins	HPLC-MS	[206]
Wheat	Bran	Phenolic acids	HPLC	[28,41]

HPLC, high-performance liquid chromatography; UPLC, ultra-performance liquid chromatography; * MS (mass spectrometry) may contemplate tandem mass spectrometry (MS*^n^*).

**Table 3:** Selected plant food by-products, screening and proposed applications.

Feedstock	Product Fraction	Evaluation Purpose and/or Application	Ref.
Almond	Skin	Effects towards antioxidant enzymes using cell and animal models.	[203]
Apple	Peel	Scavenging activity against DPPH radical, and ferric reducing antioxidant power. Inhibition of fish oil oxidation.	[151]
Avocado	Peel and seed	Reducing power (FRAP) and antioxidant potential against ABTS radical cation, DPPH radical, and reactive oxygen species (peroxyl and superoxide radical and hypochlorous acid). Anti-inflammatory activity by inhibition TNF-α and nitric oxide in mouse macrophage RAW 264.7 cells.	[11]
Barley	Outermost milling fraction	Antioxidant potential against ABTS radical cation, DPPH, peroxyl and superoxide radical. Antioxidant potential using a photoinduced chemiluminescence technique.	[204]
Barley	Outermost milling fraction	Scavenging of peroxyl and hydroxyl radicals, metal chelation activity, inhibition of radical-induced supercoiled DNA breakage and antiproliferative activities using Caco-2 human adenocarcinoma cells.	[45]
Blackberry	Seed meal	Antioxidant activity (towards hydroxyl and peroxyl radicals), reducing power, chelation capacity, prevention of DNA damage, and LDL-cholesterol oxidation.	[24]
Black raspberry	Seed	Reducing power (FRAP) and antioxidant potential towards DPPH radical and ABTS radical cation. Anti-inflammatory activity by reduction of nitric oxide using RAW 264.7 cells.	[205]
Black raspberry	Seed meal	Antioxidant activity (towards hydroxyl and peroxyl radicals), reducing power, chelation capacity, prevention of DNA damage, and LDL-cholesterol oxidation.	[24]
Blueberry	Seed meal	Antioxidant activity (towards hydroxyl and peroxyl radicals), reducing power, chelation capacity, prevention of DNA damage, and LDL-cholesterol oxidation.	[24]
Brazil nut	Skin	Antioxidant potential towards ABTS radical cation, and DPPH, hydroxyl, and peroxyl radicals.	[80]
Camelina	Seed meal	Potential biological activities of camelina and sophia seed meals through inhibition of LDL-cholesterol oxidation, DNA damage as well as pancreatic lipase and α-glucosidase activities.	[223]
Camelina	Seed meal	Antioxidant potential towards ABTS radical cation, reducing power and metal chelation.	[206]
Canola	Hull	Antioxidant potential of crude tannins by β-carotene-linoleate model system, DPPH radical, and reducing power.	[195]
Chia	Seed meal	Antioxidant potential towards ABTS radical cation, DPPH and hydroxyl radical. Reducing power, chelation capacity and antioxidant capacity in beta-carotene linoleate model system. Inhibition of activities against pancreatic lipase, α-glucosidase, human LDL-cholesterol oxidation in vitro, DNA damage induced by peroxyl and hydroxyl radicals.	[207]
*Citrus reticulata*	*Chempi* (aged peel)	Prevention of obesity and type 2 diabetes in mouse model.	[208]
Grape	Pomace	Anti-inflammatory activity in mice (inhibition of TNF-α and IL-1β).	[209]
Grape	Pomace	Antioxidant capacity using yeast cells.	[210]
Grape	Pomace	Isolation and identification of phenolics bearing inhibition capacity towards α-glucosidase.	[6]
Grape	Pomace	Antioxidant potential towards DPPH radical and ABTS radical cation.	[224]
Grape	Pomace and rachi	Antioxidant activity (towards DPPH radical, ABTS radical cation, peroxyl radical, superoxide anion, hypochlorous acid) and anti-inflammatory effect by suppressing TNF-α liberation in vitro.	[8]
Grape	Seed	Anti-inflammatory activity (inhibition of cytokines and suppression of MAPK and NF-κB) in RAW264.7 macrophages.	[225]
Grape	Seed	Reduction of bone loss in the experimental arthritis.	[226]
Grape	Seed	Reduction of kidney injury in experimental type 2 diabetes.	[227]
Grape	Winemaking by-products	Antioxidant potential towards ABTS radical cation, DPPH and hydroxyl radical. Reducing power and inhibition of α-glucosidase and lipase activities.	[30]
Grape	Winemaking and grape juice by-products	Antioxidant activity (towards DPPH radical, ABTS radical cation, and hydrogen peroxide), reducing power, prevention of DNA damage, and LDL-cholesterol oxidation.	[16]
Grape	Winemaking by-products	Bioactivity using cardiometabolic biomarkers in Wistar rats.	[15]
Guava	Pomace	Anti-inflammatory activity through reduction of edema and neutrophil migration in mice models.	[228]
Mango	Residual pulp	Microbiological safety and antioxidant activity (towards DPPH radical, ABTS radical cation)	[38]
Millet	Hull	Hydroxyl and peroxyl radical inhibition, inhibition of DNA strand scission induced by both ROS, inhibition of liposome oxidation, and human colon adenocarcinoma cell proliferation inhibition.	[171]
Onion	Skin	Inhibition of peroxyl and hydroxyl radical induced supercoiled DNA strand scission, cupric ion induced human low-density lipoprotein peroxidation inhibition in vitro, inhibition of lipopolysaccharide stimulated cyclooxygenase-2 expression in mouse macrophage cell model.	[229]
Onion	Skin	Antioxidant potential (ABTS radical cation, DPPH radical, and reducing power).	[211]
Passion fruit	Peel, albedo and seed	Microbiological safety and antioxidant activity (towards DPPH radical, ABTS radical cation)	[38]
Peanuts	Skin	Gamma-irradiation induced changes and microbiological safety. Antioxidant potential (towards DPPH radical, ABTS radical cation, hydroxyl radical, and hydrogen peroxide), reducing power, prevention of DNA damage, and LDL-cholesterol oxidation.	[17]
Peanuts	Skin and meal	Antioxidant potential against ABTS radical cation, DPPH and hydroxyl radicals, and reducing power. Antioxidant capacity in gamma-irradiated fish model system. Antimicrobial activity against Gram-positive and Gram-negative bacteria.	[12]
Peanuts	Skin and meal	Antioxidant potential towards ABTS radical cation, DPPH and hydroxyl radicals, and reducing power. Inhibition of α-glucosidase and lipase activities.	[31]
Peanuts	Skin	Isolation, structural characterization of proanthocyanidins, and evaluation of their antioxidant activity towards DPPH radical, ABTS radical cation, and ferric reducing antioxidant power.	[18]
Peanuts	Skin	Isolation and identification of proanthocyanidins. Inhibition of TNF-α and IL-6 in cultured human monocytic THP-1 cells.	[213]
Pineapple	Peel, and residual pulp	Microbiological safety and antioxidant activity (towards DPPH radical, ABTS radical cation)	[38]
Pomegranate	Peel and seed	Scavenging of ABTS radical cation, DPPH and hydroxyl radicals, and metal chelation. Potential bioactivity towards inhibition of α-glucosidase and lipase activity, inhibition of human low-density lipoprotein (LDL) oxidation in vitro and inhibition of peroxyl and hydroxyl radical-induced DNA strand scission.	[19,21]
Pomegranate	Peel and seed	Antioxidant activity in beta-carotene-linoleate model system and against DPPH radical. Prevention of lipid peroxidation in albino rat liver homogenate in vitro, scavenging activity towards hydroxyl radical scavenging activity, and human low-density lipoprotein (LDL) oxidation in vitro.	[230]
Pomegranate	Peel	Anti-inflammatory activity through inhibition of expression of TNF-α, IL-1β, MCP-1 and ICAM-1 and adhesion of monocytes to endothelial cells.	[214]
Rapeseed	Hull	Antioxidant potential of crude tannins by β-carotene-linoleate model system, DPPH radical, and reducing power.	[195]
Sophia	Seed meal	Antioxidant potential towards ABTS radical cation, reducing power and metal chelation.	[206]
Sophia	Seed meal	Potential biological activities of camelina and sophia seed meals through inhibition of LDL-cholesterol oxidation, DNA damage as well as pancreatic lipase and α-glucosidase activities.	[223]
Soybean	Seed coat	Antioxidant potential towards ABTS radical cation and DPPH as well as reducing power (FRAP assay).	[40]
Wheat	Bran	Antioxidant potential against peroxyl radical and via photochemiluminescence method, antioxidant capacity in seal blubber oil (Rancimat test) and inhibition of oxidation of low-density lipoprotein and DNA in vitro.	[41]
Wheat	Bran	Antioxidant potential against ABTS radical cation.	[42]
Wheat	Bran	Antioxidant potential against ABTS radical cation, DPPH and peroxyl radicals, reducing power, inhibition of photochemilumenescence, and iron (II) chelation activity. Inhibition of oxidation of human low-density lipoprotein cholesterol and DNA in vitro. Oxidative stability using stripped corn oil in Rancimat test.	[43]
Wheat	Bran	Antioxidant potential towards ABTS radical cation, DPPH, superoxide radicals, hydroxyl radical, and scavenging of hydrogen peroxide. Reducing power and ferrous chelating activity.	[44]
Wheat	Bran fractions	Total antioxidant capacity towards ABTS radical cation as affected by debranning.	[28]

**Table 4:** Phenolics bearing digestive enzyme inhibitory activity.

Compound	IC_50_ (µg/mL)	Ref.
A-amylase		[280]
(−)-epicatechin	140	
Epigallocatechin	>300	
(−)-4′-*O*-methylepigallocatechin	>300	
(−)-epicatechin-(4b→8)-(−)-4′-*O*-methylepigallocatechin	>300	
α-glucosidase		[280]
(−)-epicatechin	140	
Epigallocatechin	>300	
(−)-4′-*O*-Methylepigallocatechin	>300	
(−)-epicatechin-(4b→8)-(−)-4′-*O*-methylepigallocatechin	>300	
Lipase		[281]
Rosmarinic acid	125	
Chlorogenic acid	96.5	
Caffeic acid	32.6	
Gallic acid	10.1	
